# Therapy with transitions from one bone-forming agent to another: a retrospective cohort study on teriparatide and romosozumab

**DOI:** 10.1093/jbmrpl/ziae131

**Published:** 2024-10-23

**Authors:** Tomonori Kobayakawa, Yasuhide Kanayama, Yuji Hirano, Toshitaka Yukishima, Yukio Nakamura

**Affiliations:** Kobayakawa Orthopedic and Rheumatologic Clinic, Fukuroi 437-0061, Shizuoka, Japan; Department of Orthopedic Surgery and Rheumatology, Toyota Kosei Hospital, Toyota 470-0396, Aichi, Japan; Department of Rheumatology, Toyohashi Municipal Hospital, Toyohashi 441-8570, Aichi, Japan; Kobayakawa Orthopedic and Rheumatologic Clinic, Fukuroi 437-0061, Shizuoka, Japan; Department of Orthopedic Surgery, Division of Osteoporosis, Locomotive Syndrome, Joint Disease Center, Aichi Medical University, 1-1 Yazakokarimata, Nagakute, Aichi 480-1195, Japan

**Keywords:** romosozumab, teriparatide, osteoporosis, bone modeling and remodeling, sequential therapy

## Abstract

This study aimed to evaluate the effectiveness of sequential therapy with a bone formation-promoting agent (either teriparatide or romosozumab) for osteoporosis treatment following prior treatment with the other bone-forming agent (teriparatide or romosozumab). This is a multicenter retrospective cohort study observing 2 groups for comparison: one with 69 patients transitioning from teriparatide to romosozumab (the T2R group) and the other with 25 patients transitioning from romosozumab to teriparatide (the R2T group), monitored for 12 months on the second drug. Key outcomes included changes in bone mineral density (BMD), bone turnover marker changes, and adverse events. The mean ages of each group were 72.3 years in the T2R group and 67.6 years in the R2T group, with the proportions of women being 91.3% and 80.0%, respectively. The percent changes of BMD in the lumbar spine after 12 months of sequential therapy were +10.8% in the T2R group (*p* < .001 versus baseline) and −0.0% in the R2T group (*p* = .875). The percent changes in BMD in the total hip and femoral neck were +4.4% and +4.4% in the T2R group, and −1.3% and −0.8% in the R2T group, respectively. When comparing the 2 groups, BMD changes at all sites in the T2R group were significantly higher than those in the R2T group (*p* < .001). Furthermore, when examining the changes in the proportion of patients who achieved the osteoporosis treatment goal of a T-score exceeding −2.5, no significant increase was observed in the R2T group, whereas a significant increase was observed in the lumbar spine in the T2R group. Regarding therapy switching between bone-forming agents, this study suggests that transitioning from teriparatide to romosozumab increases BMD more effectively than transitioning in the opposite sequence.

## Introduction

With increasing human lifespans, the period of time required to maintain health also increases annually. It is also true for establishing the treatment strategies for osteoporosis in the long run. Currently, achieving a T-score exceeding −2.5 is suggested as an updated goal for osteoporosis treatment.^1^ To accomplish this goal, it is necessary to employ suitable treatment strategies that provide multiple agents in a suitably planned manner rather than relying on a single type of medication for a long time. In today’s practical treatment for osteoporosis with a high risk of fracture, the first-line treatment is recommended to be osteogenesis-promoting agents.^2^^,^^3^ However, the timeframe for administering bone formation-promoting agents is clearly designated: teriparatide, a form of parathyroid hormone, for up to 24 months and romosozumab for up to 12 months in Japan. These treatment proposals are generally well favored in clinical practice, with a consensus that bone formation-promoting preparations, including teriparatide and romosozumab, should be followed by bone resorption inhibitors.^2^^,^^3^ In clinical trials as well as real-world data, various studies have demonstrated significant increases in bone mineral density (BMD) following sequential therapies, ranging from teriparatide^4-7^ or romosozumab^8-10^ to antiresorptive agents, including denosumab and bisphosphonates as a standard treatment option for osteoporosis patients.

On the other hand, there are clinical scenarios that might necessitate switching from one bone formation-promoting agent to another due to an inadequate or a lack of positive therapeutic responses to the initial medication requiring further interventions, or the occurrence of adverse events. Also, it is believed that since bone formation-promoting agents are generally considered more effective at increasing BMD than bone resorption inhibitors,^11-13^ switching from one bone formation-promoting agent to another is hypothesized to yield more efficacy. Additionally, there is a critical question regarding the optimal sequencing of bone formation-promoting agents with different pharmacodynamics. In this study, we observed the impact of administering one bone-forming agent (teriparatide) followed by the other agent (romosozumab) and vice versa to increase BMD and examined the influence of their sequential administration.

## Materials and methods

**Figure 1:**
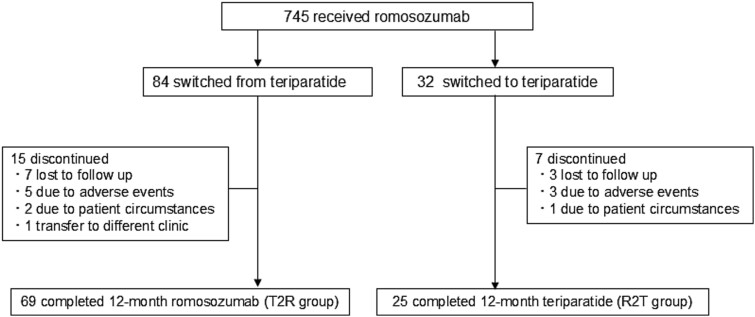
Flow diagram of the participants/patient selection throughout the study.

We retrospectively collected data from our institution and 4 collaborating facilities. Among the 745 patients with severe osteoporosis and a high fracture risk who were receiving romosozumab since March 2019, 84 had received teriparatide before romosozumab treatment (teriparatide-to-romosozumab; T2R group), and 32 had received romosozumab for 1 year followed by teriparatide (romosozumab-to-teriparatide; R2T group). Ultimately, 69 and 25 patients in the T2R and R2T groups, respectively, who completed follow-up in the switched sequential phase for 1 year were included in this study as illustrated in Figure 1. The observation period was 12 months of sequential therapy; therefore, the baseline was set as the successor drug transition point. Severe osteoporosis with a high risk of bone fragile fractures was considered an indication of lowered bone strength and was defined by meeting the following conditions: a T-score of −2.5 standard deviations (SD) or less with one or more prevalent fractures,^14^ a lumbar spine T-score of -3.3 SD or less,^15^^,^^16^ having 2 or more existing vertebral fractures,^15^^,^^17^ or evaluation as Grade 3 according to the semi-quantitative assessment method for existing vertebral fractures.^17^ All subjects fulfilled those criteria and received medications with bone formation-promoting agents covered by social health insurance. In the T2R group, patients were switched from teriparatide to romosozumab when the efficacy of teriparatide was decided to be inadequate, or when adverse events made it difficult to continue the teriparatide treatment. Conversely, in the R2T group, patients were switched to teriparatide when they were diagnosed to maintain the state of severe osteoporosis even after 12 months of romosozumab medications. In order to avoid the risk of the rapid loss of BMD when switching drugs, a washout period was not set, and the patients were transitioned to the successor drug seamlessly. The type of administering teriparatide formulations was determined at the discretion of the attending clinicians. Romosozumab was administered subcutaneously at a dose of 210 mg monthly. Teriparatide was administered subcutaneously at doses of 20 μg once daily, 56.5 μg once weekly, or 28.2 μg twice weekly.

As exclusion criteria, patients with hypercalcemia, those considered to be at high risk of developing osteosarcoma, those with primary malignant or metastatic bone tumors, or those with a history of hypersensitivity to teriparatide acetate were excluded from the study when using teriparatide products. Additionally, patients with hypocalcemia, a history of cardiovascular events, or an allergy to romosozumab at the time of administration were also excluded from the study when using romosozumab. For the patients with low serum 25-hydroxyvitamin D levels (reference range: 30-60 ng/ml), either active vitamin D3 analogs or natural vitamin D3 preparations, along with calcium supplements, were administered at the discretion of the attending clinicians.

This study was approved by the Chutoen General Medical Center Ethics Committee (Approval No. 3003231005). The study was conducted in accordance with the principles of the Declaration of Helsinki. For this type of retrospective study, formal consent was not required.

### Primary and secondary outcomes of interest

As the primary outcome of interest, BMD changes in the lumbar spine during the first year of the sequential phase were assessed and compared between both groups. Lumbar spine BMD was evaluated at all participating institutions using dual-energy X-ray absorptiometry (DXA) with a Prodigy Fuga device (GE Healthcare, Madison, WI, United States) performed by well-trained radiologists with a standard protocol. The minimum significant change observed in the model is 2%.^18^ The patients needed to have at least 2 vertebrae in the L2-L4 region and at least 1 vertebral body that could be evaluated using DXA. The lumbar spine DXA measured the L2-L4 levels and excluded any vertebral body with a T-score of 1.0 higher than that of the vertebral body with the lowest T-score. DXA results were measured at baseline and after 6 and 12 months of therapy. Similarly, the secondary endpoints included BMD changes in the total hip and femoral neck under the same conditions as the lumbar spine, as well as serum changes in bone turnover markers, including procollagen type 1 N-terminal propeptide 1 (P1NP) and tartrate-resistant acid phosphatase isoform 5b (TRACP-5b), to assess bone metabolism at baseline, 6 months, and 12 months during sequential therapy. P1NP was measured by electrochemiluminescence immunoassay (Elecsys total P1NP; Roche Diagnostics, Tokyo, Japan), with an intra- and inter-assay coefficient of variation (CV) of 2.3%-2.9% and 2.5%-4.4%, respectively. TRACP-5b was measured by enzyme-linked immunosorbent assay (Osteolinks-TRACP-5b; Nitto Boseki, Koriyama, Japan) with an intra- and inter-assay CV of 2.3%-3.7% and 1.8%-2.9%, respectively. The TRACP-5b level is a useful bone resorption marker with higher clinical sensitivity and signal-to-noise ratios than other serum markers including carboxy-terminal collagen crosslink levels.^19^ Moreover, through 1 year of observation, we monitored the changes in the proportion of patients who achieved a T-score over −2.5 as a treatment target from the start of sequential therapy to 6 and 12 months thereafter. Additionally, we recorded adverse events during treatment to assess drug safety.

### Statistical analysis

Patient background parameters and percent changes in BMD are expressed as mean ± standard deviation, while P1NP and TRACP-5b levels are expressed as median [interquartile range]. Using the Wilcoxon signed-rank test, percent changes from baseline to 6 and 12 months for BMD, P1NP, and TRACP-5b were assessed. The Wilcoxon rank-sum test evaluated group differences in percent changes from baseline. Fisher exact test determined study group characteristic differences. Also, McNemar test was employed to determine differences in a paired set of marginal frequencies. Significance was set at *p* < .05. All statistical tests were conducted using EZR^20^ (version 1.52; Saitama Medical Center, Jichi Medical University, Saitama, Japan), a graphical user interface for R (The R Foundation for Statistical Computing, Vienna, Austria). This interface is a modified version of the R commander designed to add statistical functions frequently used in biostatistics.

## Results

We observed 69 patients in the T2R group, and 25 in the R2T group. In the T2R group, the reasons to switch to romosozumab included 59 cases of inadequate efficacies, 5 cases of adverse events, and 5 cases of patients’ circumstances. In the R2T group, all subjects switched to teriparatide soon after the completion of a series of 12-month romosozumab administration since they were diagnosed to maintain a state of severe osteoporosis even after romosozumab administration. Regarding the patient background of each group at baseline, the time of initiation of the second agent, the mean age of the patients in the T2R group was 72.3 ± 12.2 years, with 63 female patients (91.3%). In particular, the average duration of teriparatide before romosozumab in the T2R group was 19.6 ± 5.3 months, and the detailed preparations of teriparatide included subcutaneous injections of 20 μg daily (49 cases), 56.5 μg every single week (12 cases), and 28.2 μg twice a week (4 cases). The T-scores were − 2.88 ± 1.29, −2.64 ± 0.83, and − 3.06 ± 0.99 for the lumbar spine, total hip, and femoral neck, respectively. Similarly, the R2T group had a mean age of 67.6 ± 10.6 years and included 20 female patients (80.3%). The T-score was −1.32 ± 1.52, −1.97 ± 0.68, and − 2.36 ± 0.73 for the lumbar spine, total hip, and femoral neck, respectively (Table 1). The T-scores of the T2R group were significantly lower than those of the R2T group in all areas (all *p* < .01). Furthermore, the prevalence of previous vertebral fractures was significantly higher in the R2T group (96%) than that in the T2R group. Regarding bone metabolism, the T2R group demonstrated significantly enhanced bone formation and resorption compared with that of the R2T group. Specifically, P1NP levels were notably higher in the T2R group (78.8 μg/L [49.3, 132.7]) than in the R2T group (46.2 μg/L [35.8, 58.9]) (*p* < .01). Furthermore, TRACP-5b levels were 463.0 mU/dL (367.3, 833.5) in the T2R group and 235.5 mU/dL (192.5, 298.3) in the R2T group (*p* < .001).

**Table 1:** Baseline patient characteristics of the 2 groups.

	T2R (*n* = 69)	R2T (*n* = 25)	*p*-value
**Age (years)**	72.3 ± 12.2	67.6 ± 10.6	0.092
**Female, n (%)**	63 (91.3)	20 (80.0)	0.154
**BMI (kg/m^2^)**	20.3 ± 3.3	21.3 ± 3.7	0.225
**Duration of prior treatment (M)**	17.4 ± 8.8	12.0 ± 0.0	<0.001
**Type of osteoporosis Primary**	40 (58.0)	12 (48.0)	0.483
**Secondary**	29 (42.0)	13 (52.0)	
**BMD T-score**			
**Lumbar spine**	−2.88 ± 1.29	−1.32 ± 1.52	<0.001
**Total hip**	−2.64 ± 0.83	−1.97 ± 0.68	<0.01
**Femoral neck**	−3.06 ± 0.99	−2.36 ± 0.73	<0.01
**Prior vertebral fracture, n (%)**	34 (49.3)	24 (96.0)	<0.001
**Prior non-vertebral fracture, n (%)**	21 (30.4)	8 (32.0)	1.000
**Combined use of active Vit D, n (%)**	45 (65.2)	13 (52.0)	0.161
**PINP (μg/L, median [IQR])**	78.8 [49.3, 132.7]	46.2 [35.8, 58.9]	<0.01
**TRACP-5b (mU/dL, median [IQR])**	463.0 [367.3, 833.5]	235.5 [192.5, 298.3]	<0.001
**Serum albumin (g/dL)**	4.1 ± 0.3	4.2 ± 0.3	0.160
**Serum corrected calcium (mg/dL)**	9.3 ± 0.3	9.3 ± 0.4	0.821
**eGFR (mL/min/1.73 m^2^)**	71.1 ± 19.5	67.1 ± 18.4	0.379
**25OHD (ng/mL)**	17.5 ± 10.8	15.2 ± 4.4	0.410

Patients’ characteristics at baseline (the time of initiation of second drug). Data are expressed as mean ± standard deviation or number (%) of all patients who completed the 12-month treatment with either teriparatide or romosozumab as second agents. P1NP and TRACP-5b levels are expressed as median values [IQRs].

Group differences were analyzed using Student t-test, Wilcoxon rank-sum test, or Fisher exact test.

Abbreviations: BMI, body mass index; BMD, bone mineral density; IQR, interquartile range; P1NP, procollagen type 1 N-terminal propeptide; TRACP-5b, tartrate-resistant acid phosphatase isoform 5b; eGFR, estimated glomerular filtration rate; 25OHD, 25-hydroxyvitamin D

Table 2 represents the patients’ characteristics before the initiation of the first bone formation-promoting agent. Although 2 group showed similar characteristics, the T2R group exhibited significantly lower T-scores of the lumbar spine compared with the R2T group, and a higher proportion of patients in the T2R group with a history of prior vertebral fractures.

**Table 2:** Patient characteristics before the initiation of the first treatment phase.

	T2R (*n* = 69) (Teriparatide)	R2T (*n* = 25) (Romosozumab)	*p*-value
**BMD T-score**			
**Lumbar spine**	−2.94 ± 1.16	−2.25 ± 1.21	<0.05
**Total hip**	−2.47 ± 0.85	−2.22 ± 0.87	0.235
**Femoral neck**	−2.80 ± 1.07	−2.67 ± 1.03	0.603
**BTM**			
**P1NP (μg/L, median [IQR])**	67.4 [45.8, 105.2]	48.8 [24.8, 78.0]	0.077
**TRACP-5b (mU/dL, median [IQR])**	456.5 [360.3, 718.0]	416.0 [275.8, 527.0]	0.110
**Prior vertebral fracture, n(%)**	32(46.4)	24 (96.0)	<0.001
**Prior non-vertebral fracture, n(%)**	17(24.6)	7 (28.0)	0.791

Data are expressed as mean ± standard deviation or number (%) of all patients who completed the 12-month treatment with either teriparatide or romosozumab. P1NP and TRACP-5b levels are expressed as median values [IQRs].

Group differences were analyzed using Student t-test, Wilcoxon rank-sum test, or Fisher exact test.

Abbreviations: BMD, bone mineral density; BTM, bone turnover marker; IQR, interquartile range; P1NP, procollagen type 1 N-terminal propeptide; TRACP-5b, tartrate-resistant acid phosphatase isoform 5b

### Primary outcome of interest

**Figure 2:**
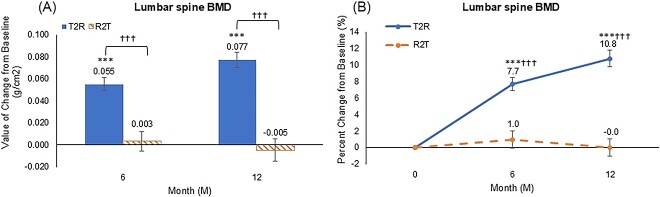
Mean changes in bone mineral density at lumbar spine (A) value of change (B) percent change, data expressed as mean values, and bars indicate mean ± standard error. ^*^p < .05, ^*^^*^p < .01, and ^*^^*^^*^p < .001 versus baseline (Wilcoxon signed-rank test). †*p* < .05, ††*p* < .01, and †††*p* < .001 versus the romosozumab group (Wilcoxon rank-sum test), T2R: teriparatide-to-romosozumab; R2T: romosozumab-to-teriparatide.

Figure 2 illustrates the BMD changes in the lumbar spine among each group. In the T2R group, BMD increased by +0.055 g/cm^2^ ± 0.047 (+7.7% ± 6.2%) at 6 months and by +0.077 g/cm^2^ ± 0.054 (+10.8% ± 7.7%) at 12 months (*p* < .001 versus baseline). However, BMD changed by +0.003 g/cm^2^ ± 0.043 (+1.0% ± 5.2%) at 6 months and  − 0.005 g/cm^2^ ± 0.050 (−0.0% ± 5.1%) in the R2T group, with no significant difference from baseline (*p* = .710 at 6 months and *p* = .875 at 12 months). Statistical analysis revealed that BMD in the T2R group was significantly higher than that in the R2T group at 6 and 12 months (*p* < .001).

### Secondary outcome of interest

**Figure 3:**
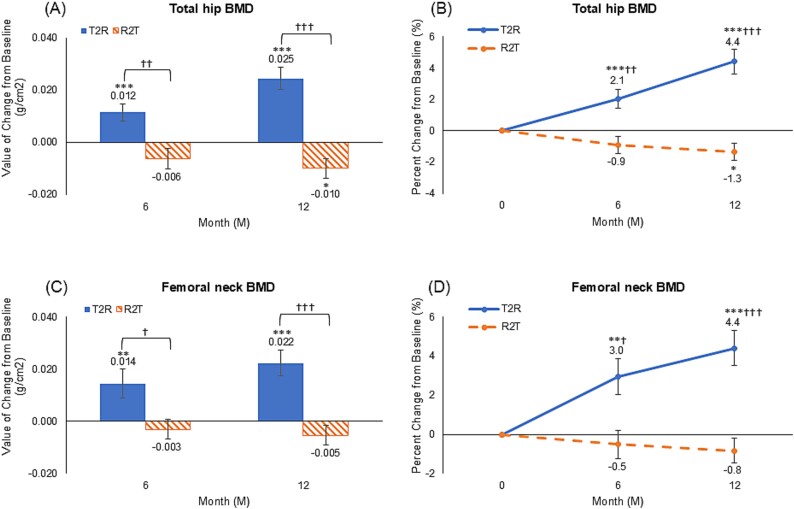
Mean values and percent changes in bone mineral density at total hip (A, B) and femoral neck (C, D), data expressed as mean values and bars indicate mean ± standard error. ^*^p < .05, ^*^^*^p < .01, and ^*^^*^^*^p < .001 versus baseline (Wilcoxon signed-rank test). †*p* < .05, ††*p* < .01, and †††*p* < .001 versus the romosozumab group (Wilcoxon rank-sum test), T2R: teriparatide-to-romosozumab; R2T: romosozumab-to-teriparatide.

Figure 3 describes the BMD changes in the total hip and femoral neck. Regarding total hip BMD (Figure 3A and B), the changes in the T2R group were + 0.012 g/cm^2^ ± 0.027 (+2.1% ± 4.9%; *p* < .001 versus baseline) at 6 months and + 0.025 g/cm^2^ ± 0.036 (+4.4% ± 6.5%; *p* < .001) at 12 months, and −0.006 g/cm^2^ ± 0.019 (−0.9% ± 2.7%; *p* = .260) at 6 months and − 0.010 g/cm^2^ ± 0.018 (−1.3%  ± 2.6%; *p* < .05) at 12 months in the R2T group. Regarding femoral neck changes at 6 and 12 months (Figure 3C and D), the changes were +0.014 g/cm^2^ ± 0.045 (+3.0% ± 7.3%; *p* < .01) and +0.022 g/cm^2^ ± 0.040 (+4.4% ± 7.1%; *p* < .001) in the T2R group, and −0.003 g/cm^2^ ± 0.023 (−0.5% ± 3.4%; *p* = .543) and −0.005 g/cm^2^ ± 0.020 (−0.8% ± 3.0%; *p* = .339) in the R2T group, respectively. In the T2R group, the BMD at both sites was significantly increased compared with that at baseline (*p* < .001). However, BMD did not change significantly from baseline at most measurement points in the R2T group, except for the total hip BMD at 12 months, revealing a significant decrease. Significant differences were observed between the groups throughout the study period (at 6 and 12 months, the *p*-values for the total hip were *p* < .01 and *p* < .001, and those for the femoral neck were *p* < .05 and *p* < .001, respectively), and those changes exhibited a similar trend at the lumbar spine.

**Figure 4:**
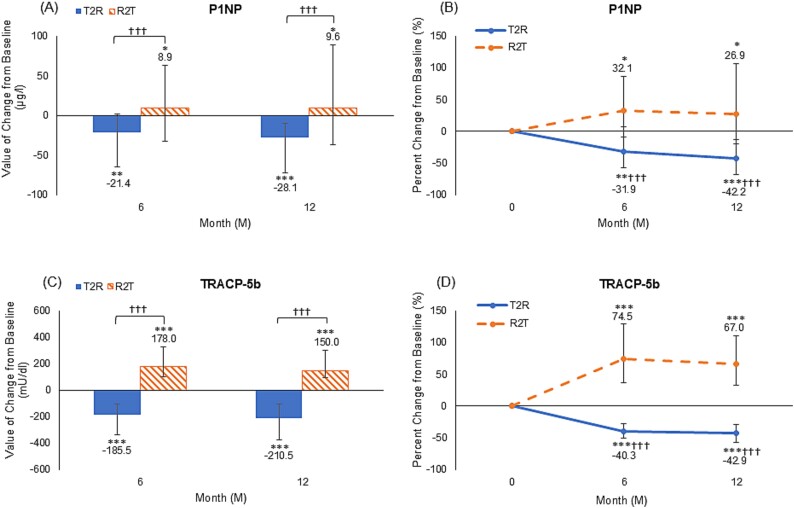
Median values and percent changes in P1NP (A and B) and TRACP-5b (C and D), bars indicate the interquartile range. ^*^p < .05, ^*^^*^p < .01, and ^*^^*^^*^p < .001 versus baseline (Wilcoxon signed-rank test). †*p* < .05, ††*p* < .01, and †††*p* < .001 versus the romosozumab group (Wilcoxon rank-sum test), P1NP, procollagen type 1 N-terminal propeptide 1; TRACP-5b, tartrate-resistant acid phosphatase isoform 5b, T2R: teriparatide-to-romosozumab; R2T: romosozumab-to-teriparatide.

Furthermore, as for the median serum bone turnover maker changes [interquartile range], significant changes in P1NP were observed at 6 and 12 months (Figure 4A and B), with values of −21.4 μg/L [−64.5, +1.8] (−31.9% [−57.4, +7.8]; *p* < .01 versus baseline) and − 28.1 μg/L [−71.9, −9.5] (−42.2% [−67.8, −12.5]; *p* < .001) in the T2R group, while those of the R2T group were +8.9 μg/L [−4.8, +29.5] (+32.1% [−9.5, +86.1]; *p* < .05) and +9.6 μg/L [−6.1, +47.3] (+26.9% [−19.3, +106.5]; *p* < .05), respectively. Significant differences existed between the groups at 6 and 12 months (*p* < .001). Similarly, median values of TRACP-5b changes at 6 and 12 months were −185.5 mU/dL [−333.3, −107.0] (−40.3%[−50.3, −27.2]) and −210.5 mU/dL [−376.8, −104.5] (−42.9% [−57.5, −29.2]) in the T2R group, and +178.0 mU/dL [+103.0, +326.0] (+74.5% [+36.7, 129.6]) and +150.0 mU/dL [+94.0, +300.0] (+67.0% [+32.4, +110.7]) in the R2T group, respectively (Figure 4C and D). Furthermore, there were significant differences when comparing values from baseline as well as when comparing between the 2 groups (all *p* < .001). For both markers, the 2 groups demonstrated significantly contrasting results.

**Figure 5:**
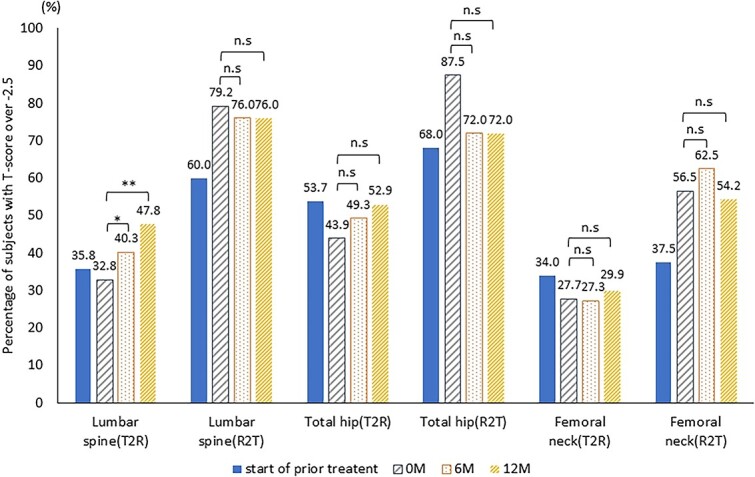
Proportion of participants with T-scores over −2.5 throughout the study n.s.; no significance, ^*^p < .05, ^*^^*^p < .01 versus baseline (McNemar test). T2R: teriparatide-to-romosozumab; R2T: romosozumab-to-teriparatide.

Next, Figure 5 illustrates the proportions of patients who had T-scores exceeding −2.5 in each group at the time of each observation; before the administration of the first bone formation-promoting drug and the observation points of the second drug (0, 6, and 12 M). In the T2R group, the proportion of high T-score did not seem to increase significantly after the use of teriparatide; however the proportions were increased throughout the study with romosozumab administration, and especially in the lumbar spine, the increase was statistically significant: *p* < .05 at 6 months, and *p* < .01 at 12 months versus baseline (total hip, *p* = .371 at 6 months and *p* = .114 at 12 months; femoral neck, *p* = 1.000 at both 6 and 12 months). In contrast, in the R2T group, there was no significant change in the proportions even with sequential therapy with teriparatide (lumbar spine, *p* = 1.000 at both 6 and 12 months; total hip, *p* = .248 at both 6 and 12 months; and femoral neck, *p* = .617 at 6 months and *p* = 1.000 at 12 months).

### Safety assessment

Table 3 summarizes the adverse events during the study period. In the T2R group, there were 5 severe adverse event cases causing treatment discontinuation, including cerebral infarction, cerebral hemorrhage, and one new fracture. Three patients in the R2T group experienced nausea and discomfort. Furthermore, we reported 12 injection site reactions and 4 other events in the T2R group and 5 other events in the R2T group; however, these were relatively mild symptoms with no obstacles to continuing treatment. A significantly higher prevalence of injection site reactions was noted in the T2R group, while there was a greater prevalence of nausea in the R2T group. There was no significant difference in the overall prevalence of adverse events between the groups (*p* = .814).

**Table 3:** Adverse events.

	T2R (*n* = 84)	R2T (*n* = 32)	*p*-value
**Overall adverse events**	21 (25.0)	8 (25.0)	0.814
**Severe adverse events leading to study discontinuation**	5 (6.0)	3 (9.4)	1.000
**Cerebral infarction**	1 (1.2)	0	1.000
**Cerebral hemorrhage**	1 (1.2)	0	1.000
**Supracondylar femur fracture**	1 (1.2)	0	1.000
**Headache**	1 (1.2)	0	1.000
**Arrhythmia**	1 (1.2)	0	1.000
**Nausea/Discomfort**	0	3 (9.4)	0.017^*^
**Injection site reactions**	12 (14.3)	0	0.035^*^
**Pain**	9 (10.7)	0	0.110
**Swelling**	3 (3.6)	0	0.566
**Other events of interest**	4 (4.8)	5 (15.6)	0.052
**Fatigue**	2 (2.4)	0	1.000
**Eczema**	1 (1.2)	0	1.000
**Hypocalcemia**	1 (1.2)	0	1.000
**Nausea/Discomfort**	0	1 (3.1)	1.000
**Stomach upset**	0	1 (3.1)	1.000
**Cold sweat**	0	1 (3.1)	1.000
**Blood pressure elevation**	0	1 (3.1)	1.000
**Swelling**	0	1 (3.1)	1.000

Data are expressed as number (%) of patients. Group differences were analyzed using Fisher exact test. ^*^p < .05

Injection site reactions included adverse events on the skin at the injection site that lasted for 2 days or longer.

## Discussion

The 12-month observation of romosozumab in patients transitioning from teriparatide demonstrated significantly greater efficacy in increasing BMD in the lumbar spine, total hip, and femoral neck compared with the 12-month observation of teriparatide transitioning from romosozumab. Previous studies have verified that the effectiveness of osteoporosis medications is significantly influenced by the sequence in which they are administered as well as the types of treatment action; osteogenic or antiresorptive. For example, sequential treatment from teriparatide to denosumab was demonstrated to be effective in a DATA study, whereas denosumab to teriparatide treatment resulted in a temporary BMD decrease.^4^ Furthermore, a history of treatment with bisphosphonate agents or denosumab has been reported to reduce teriparatide^21^ and romosozumab^8^^,^^22^^,^^23^ efficacy compared with the treatment-naïve group. In other words, the order of osteoporosis medications requires careful consideration, with proper management to realize better treatment effectiveness. In this study, the T2R group exhibited a lower T-score at the time of transition, while the R2T group had a higher T-score with significant differences. Although both teriparatide^24^^,^^25^ and romosozumab^12^^,^^26^ are well known to significantly increase BMD, particularly in the lumbar spine, the T-score remained lower in the T2R group than the R2T group throughout the study. This suggests that switching to romosozumab would result in a significant increase in BMD, particularly when the effect of teriparatide on BMD is insufficient. Conversely, in the R2T group, the administration of teriparatide was unlikely to further increase BMD when BMD had already been substantially improved by the prior treatment with romosozumab.

Transition therapy switching from teriparatide to romosozumab was discussed in a previous study to demonstrate its efficacy,^8^^,^^27^^,^^28^ and our study had similar results of increased BMD.

Regarding the dynamics of bone metabolism markers, both bone formation and bone resorption markers decreased in the T2R group; however, the rate of decrease in a bone resorption marker was greater than that of a bone formation marker, suggesting the effective formation of an anabolic window, which likely contributed to the observed remarkable increase in BMD. The action of teriparatide results in the powerful acceleration of both bone formation and resorption, and the discontinuation of teriparatide might lead to a halt of the action. Consequently, both bone formation and resorption are rapidly diminished upon discontinuation. On the other hand, although romosozumab also has pronounced bone formation-promoting effects, the remarkable decrease in a bone formation marker due to the discontinuation of teriparatide was superior to the impact of romosozumab. Simultaneously, the discontinuation of teriparatide also resulted in a similar reduction in a bone resorption marker. This reduction in cooperation with the bone resorption-suppressing effect of romosozumab results in a significant increase in a bone resorption marker, suggesting that the reduction in bone resorption surpassed the reduction in bone formation, thereby creating an anabolic window. The dynamics of bone metabolism markers during the sequential therapies from teriparatide to romosozumab align with findings from previous studies,^22^^,^^23^ further supporting our verifications.

However, when focusing on patients who switched from romosozumab to teriparatide, romosozumab discontinuation is presumed to result in a higher bone hypermetabolic turnover state in bone formation and resorption before starting osteoporosis treatment. Furthermore, the discontinuation might trigger the suppression of osteoprotegerin inhibition, causing transient mature osteoclast proliferation leading to bone resorption acceleration, as frequently seen in cases of denosumab discontinuation.^29-32^ Administering teriparatide in these conditions enhances bone resorption, followed by bone formation. However, bone resorption enhancements exceed that of bone formation, resulting in an imbalance of bone metabolism and a lack of anabolic window formation. Consequently, no significant BMD increases were observed. Furthermore, if the existing teriparatide still absorbs old bone, there may be a limited amount of bone undergoing refreshment through bone metabolism. This is because romosozumab actively promotes new bone structure formation, contributing to the lack of a substantial BMD increase.

Furthermore, with regard to patients who had T-scores exceeding −2.5, no significant increase in the proportion was found in the R2T group; on the other hand, a significant increase in the lumbar spine T-score was observed in the T2R group. In this study, in cases where the positive response to teriparatide is inadequate, romosozumab has seemed to be an effective treatment. However, after achieving a certain level of improvement in BMD with romosozumab, switching from romosozumab to teriparatide did not result in a further increase in BMD, despite the intention to enhance fracture prevention. Furthermore, among the patients with severe osteoporosis at a high risk of fragility fracture, the proportions of those with a T-score of −2.5 or greater especially at total hip and femoral neck was clearly higher when romosozumab was used first, and this advantage was not reversed when switching from teriparatide to romosozumab. Therefore, if the treatment aims to achieve a more significant increase in BMD, it is possibly preferable to select romosozumab as an initial treatment.

Regarding the incidents of adverse events, the T2R group primarily exhibited minor injection site reactions. Conversely, in the R2T group, there were some cases leading to treatment discontinuation including nausea and discomfort, indicating that cautions and observations should be exercised when using teriparatide by the attending clinicians.

The limitations of this study include (1) a small sample size; (2) the 2 drugs had different designated durations: the observation period of romosozumab was arranged to 1 year, but the duration of prior teriparatide varied and was not standardized for observation, as shown above; therefore, we observed only 1 year of the subsequent administration phase for fair direct comparison; (3) no subcategorized analysis of teriparatide preparations into daily, twice-weekly, and once-weekly subcutaneous injections; and (4) the influence of the prior-to-previous medication (in the R2T group, the medication before romosozumab use, and in the T2R group, the medication before teriparatide initiation), which was not taken into account. However, because the prior-to-previous medication was used for more than 2 years before the data collection period of this study, its impact was considered minimal.

## Conclusion

This study investigated the impact of sequential therapy with bone formation-promoting agents, (teriparatide and romosozumab), following one another, revealing which agent should be preceded for increased BMD in patients with severe osteoporosis. Our data demonstrated that sequential therapy from teriparatide to romosozumab exhibited greater benefits in increasing BMD at any site rather than switching from romosozumab to teriparatide.

## Data Availability

The datasets analyzed and presented in this study are available from the corresponding author upon reasonable request.
